# Prognostic value of high FoxC2 expression in resectable non-small cell lung cancer, alone or in combination with E-cadherin expression

**DOI:** 10.1186/s12885-016-2056-0

**Published:** 2016-01-13

**Authors:** Wei Jiang, Hong Fan, Cheng Qian, Jianyong Ding, Qun Wang, Xuguang Pang

**Affiliations:** Department of Thoracic Surgery, Zhongshan Hospital, Fudan University, 180 Fenglin Road, Xuhui District, Shanghai, 200032 China

**Keywords:** Lung cancer, FoxC2, E-cadherin, Prognosis, Immunohistochemistry

## Abstract

**Background:**

FoxC2 is an epithelial–mesenchymal transition (EMT) regulator which induces metastasis. The purpose of this study is to assess the prognostic value of FoxC2 expression in non-small cell lung cancer (NSCLC), alone or in combination with E-cadherin expression.

**Methods:**

A retrospective study was conducted using immunohistochemistry to investigate FoxC2 and E-cadherin expression in a cohort of 309 patients with surgically resected NSCLCs. The prognostic value of FoxC2 and E-cadherin on overall survival (OS) and recurrence-free survival (RFS) was determined by Kaplan-Meier analysis and Cox proportional hazard models.

**Results:**

High FoxC2 expression was detected in 26.5 % of tumors, and significantly correlated with tobacco use (*p* = 0.047), adenocarcinoma (*p* = 0.008) and nodal involvement (*p* < 0.001). Univariate analysis revealed its association with OS (*p* = 0.036) and RFS (*p* = 0.011). By multivariate analysis, high FoxC2 expression lost its significance as an independent predictor of recurrence (*p* = 0.077), while TNM stage, nodal status and the presence of high FoxC2 and impaired E-cadherin expression retained independent prognostic significance in relation to both OS and RFS. Subset analyses indicated that high FoxC2 expression was significantly associated with disease outcome in node-positive, but not in node-negative patients.

**Conclusion:**

Evaluation of FoxC2 expression, alone or in combination with E-cadherin expression, may help to stratify NSCLC patients for risk of disease progression, pointing to this EMT regulator as a potential prognostic marker.

## Background

Non-small cell lung cancer (NSCLC) is the leading cause of cancer-related mortality worldwide. Despite recent therapeutic advances, the 5-year survival rate across all stages of this malignancy is approximately 15 %, as the majority of patients present at the diagnosis with advanced disease [1]. Although it is surgically curable when diagnosed at early stage, metastasis remains the major obstacle to long-term survival after surgical resection [2]. However, conventional staging parameters, such as the tumor/node/metastasis (TNM) system, fail to provide precise risk stratification that can identify patients more likely to recur and have poor prognoses. Thus, there is an urgent need for the identification of new and more reliable prognostic markers and novel therapeutic targets. Given the significant impact of metastasis on survival, metastasis-related molecules may have this potential.

Epithelial-to-mesenchymal transition (EMT) is a process by which cells undergo a developmental switch from an epithelial to a motile mesenchymal phenotype [3]. Essential for the development of embryonic mesoderm, EMT is also considered to be one of the crucial molecular mechanisms inducing tumor invasion and metastasis [4, 5]. Loss of E-cadherin expression and the subsequent reduction of the ability of cells to form stable cell-cell contacts is a hallmark of EMT [3]. Several transcription factors, such as the basic helix-loop-helix protein Twist, the zinc-finger proteins Snail and Slug, the E-box-binding protein ZEB1 and the forkhead box protein FoxC2 has been reported to induce EMT through the repression of E-cadherin expression, thereby playing pivotal roles in tumor metastasis [6–9].

FoxC2 is a member of the forkhead transcription factor family and an important regulator of lymphovascular vessel formation in cardiovascular development and disease [10]. Recent studies suggest that FoxC2 is an EMT inducer and correlates with tumor metastasis and angiogenesis [9, 11, 12]. Furthermore, an in vitro study demonstrated that FoxC2 lies at the crossroads of EMT and cancer stem cell properties in breast cancer [13]. However, despite all these biochemical and functional findings, the prognostic role of FoxC2 in cancers has not been extensively studied, especially in the context of lung cancer. Previously, we showed that a three-marker model including FoxC2 accurately predicted survival of stage I NSCLC by using immunohistochemistry in tissue microarrays of 137 cases [14]. This result prompted us to investigate FoxC2 expression and assess its prognostic value in a larger clinical cohort of patients with NSCLC.

The aims of this study were: (1) to examine the expression of FoxC2 in surgically resected NSCLC and correlate it with clinicopathologic characteristics commonly related to disease prognosis, in a cohort of 309 patients; (2) to analyze its prognostic significance in relation to clinical outcome, using subset analyses; (3) to determine the prognostic impact of FoxC2 expression by multivariate analysis, either as an independent parameter or in combination with E-cadherin; (4) to identify subsets of patients with undesirable outcomes after prognostic stratification based on the expression levels of these two markers. We suggest that FoxC2 may have a role in promoting NSCLC invasiveness and is a promising independent predictor for recurrence and survival.

## Methods

### Patients and specimens

Archival formalin-fixed, paraffin-embedded specimens from surgically resected NSCLC containing tumor and adjacent normal tissues were collected from 309 patients at Zhongshan Hospital between 2006 and 2010. Informed consent was obtained, and this study was approved by the ethics committee of Zhongshan Hospital. All patients underwent lobectomy or pneumonectomy with mediastinal lymph node dissection. Cases treated preoperatively with chemo-and/or radiotherapy were excluded. Detailed information about patient demographics, clinical manifestation and histopathology was collected retrospectively for all patients. Histologic classification of tumors was based on the World Health Organization criteria. Tumor stage was determined according to the TNM 7th edition of International Union Against Cancer.

### Follow-up and postoperative treatment

Criteria for adjuvant therapy included advanced disease (chemotherapy) and better local disease control (radiotherapy). The median follow-up period was 49 (range, 3 to 81) months, lasting through October 30, 2012. All patients were followed up quarterly in the first 2 years and semi-annually thereafter. The treatment modality after relapse varied among individuals, and 11 patients who recurred received no treatment. The primary end point was overall survival (OS) as measured from the date of surgery to the date of death or last contact. Recurrence-free survival (RFS) was calculated as the period from surgery until the time of recurrence.

### Tissue microarray and immunohistochemistry

All samples from patients with NSCLC were stained with hematoxylin and eosin. Duplicates of 1 mm diameter cores from the tumor center and noncancerous margin (2 punches, designated as tumor and corresponding normal tissue, respectively) were included in each sample, along with controls, to ensure reproducibility and homogenous staining. Two tissue microarray blocks were constructed, each containing 315 cores. Sections 4-μm thick were placed onto slides coated with 3-aminopropyltriethoxysilane. The primary antibodies were as follows: mouse monoclonal antibody against FoxC2 (ab55004; Abcam, Cambridge, UK) diluted 1:100, and mouse monoclonal antibody against E-cadherin (ab1416; Abcam, Cambridge, UK) diluted 1:100. Immunohistochemistry of paraffin sections was performed using a two-step protocol (Novolink Polymer Detection System; Novocastra, Newcastle, UK) according to the manufacturer’s instructions. Briefly, paraffin sections were deparaffinized and hydrated. After microwave antigen retrieval, endogenous peroxidase activity was blocked by incubation of slides in 0.3%H_2_O_2_, and nonspecific binding sites were blocked with Protein Block (RE7102; Novocastra). After serial incubation with primary antibodies, Post Primary Block (RE7111; Novocastra), and secondary antibody (Novolink Polymer RE7112; Novocastra), the sections were developed in diaminobenzidine solution under a microscope and counterstained with hematoxylin. Negative control slides that omitted the primary antibodies were included in all assays.

### Evaluation of immunohistochemistical staining

All samples were evaluated by two pathologists who were blinded to the patients’ outcome. When a discrepancy was found, consensus was reached using simultaneous examination by all two investigators. The level of immunoreactivity of FoxC2 was graded according to the percentage of positively stained tumor cells (0, no staining; 1, 1–30 %; 2, 30–60 %; 3, >60 %). Only a grade of greater than 3 was considered as a positive immunohistochemistry result (high expression). The intensity of staining was carefully determined and assessed using a 4-grade scale as well (0, no staining; 1, weak staining; 2, moderate staining; 3, strong staining). A grade of greater than 2 was considered as positive staining. Staining for E-cadherin was graded by 3° compared to the staining intensity of positive control core specimens: grade 0 (no expression or minimal expression with less than 10 % of tumor cells stained), grade 1 (reduced expression or heterogeneous expression), and grade 2 (preserved expression with more than 90 % of the tumor cells stained). For the statistical analysis, expression was grouped into two categories: 0 and 1, impaired expression; 2, preserved expression.

### Statistical analysis

Associations between clinicopathologic characteristics and protein expression intensities were evaluated with the chi-square test. OS and RFS were calculated by the Kaplan–Meier method and were analyzed by the log-rank test. Univariate and multivariate analyses were based on the Cox proportional hazards regression model for independent prognostic value. SPSS 15.0 statistical software (SPSS, Chicago, IL, USA) was used. A two-tailed p-value of less than 0.05 was considered statistically significant.

## Results

### Pattern of FoxC2 expression in NSCLC and correlation to clinicopathological parameters

**Fig. 1:**
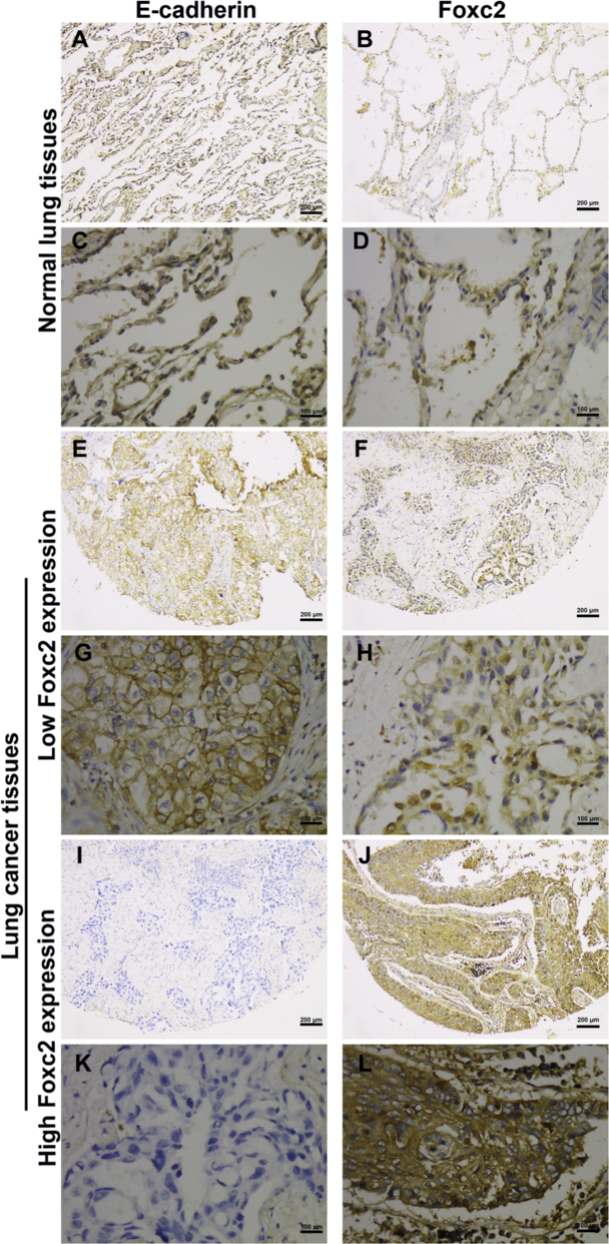
Representitive examples of FoxC2 and E-cadherin expression in NSCLC and normal lung tissue. Immunoreactivity of FoxC2 was mainly detected in the cytosol and nuclei, while E-cadherin expression was primarily membranous. High level of FoxC2 expression was correlated with impaired E-cadherin expression. **a** and **c** preserved expression of E-cadherin in normal tissue; (**b** and **d**) weak expression of FoxC2 in normal tissue; (**e** and **g**) preserved expression of E-cadherin in NSCLC; (**f** and **h**) low expression of FoxC2 in NSCLC; (**i** and **k**) impaired expression of E-cadherin in NSCLC; (**j** and **l**) high expression of FoxC2 in NSCLC

Immunostaining of FoxC2 generally displayed a nucleoplasmic pattern, with a tendency for cytoplasmic localization. In normal bronchial and alveolar epithelial cells, FoxC2 staining was very weak and nearly negative in some cases. On the other hand, increased FoxC2 staining was observed in NSCLC tissues, with 82 (26.5 %) tumors showing a high level of expression. Representative images are shown in Fig. 1. None of cells morphologically exhibited a spindle-cell transdifferentiation that is characteristic of EMT.

Correlations of FoxC2 expression with clinicopathologic parameters are shown in Table 1. FoxC2 expression showed a significant correlation with tobacco use (*p* = 0.047) and pN status (*p* < 0.001). Notably, high FoxC2 expression correlated with the histologic type of adenocarcinoma (*p* = 0.008). No significant associations were noted between FoxC2 expression and other clinicopathologic variables.

**Table 1:** Correlations between staining of Foxc2, E-cadherin and clinicophathologic characteristics in 309 NSCLC patients

Variables	Foxc2-L	Foxc2-H	*p*	E-cadherin-I	E-cadherin-P	*p*
	*n* = 227	*n* = 82		*n* = 127	*n* = 182	
Age						
≤ 65	89	26	0.062	45	70	0.551
> 65	138	56		82	112	
Sex						
Male	154	55	0.891	90	119	0.329
Female	73	27		37	63	
Tobacco use						
No	117	35	0.047	59	93	0.223
Yes	110	47		68	89	
Pathological type						
Adenocarcinoma	131	58	0.008^b^	77	112	0.653^b^
Squamous cell carcinoma	76	13		40	49	
Other^a^	20	11		10	21	
TNM stage						
I	45	17	0.679	22	40	0.432
II–III	182	65		105	142	
pT status						
T1	57	18	0.549	33	42	0.516
T2-4	170	64		94	140	
pN status						
N0	79	12	< 0.001	30	61	0.041
N1-2	148	70		97	121	
Pleural involvement						
No	181	68	0.372	98	151	0.245
Yes	46	14		29	31	
Differentiation						
Well/moderate	85	33	0.606	45	73	0.338
Poor	142	49		82	109	
Vascular invasion						
No	200	71	0.617	109	162	0.568
Yes	27	11		18	20	

*FoxC2-H* high expression of FoxC2; *FoxC2-L* low expression of FoxC2

*E-cadherin-I* impaired expression of E-cadherin; *E-cadherin-P* preserved expression of E-cadherin

^a^ Other including adenosquamous carcinoma, mucoepidermoid carcinoma, carcinosarcoma, large-cell carcinoma, and atypical carcinoid

^b^ *p* value was analyzed by Adenocarcinoma vs. non- Adenocarcinoma using the chi-square test

### High expression of FoxC2 as a prognostic factor in patients with NSCLC

**Fig. 2:**
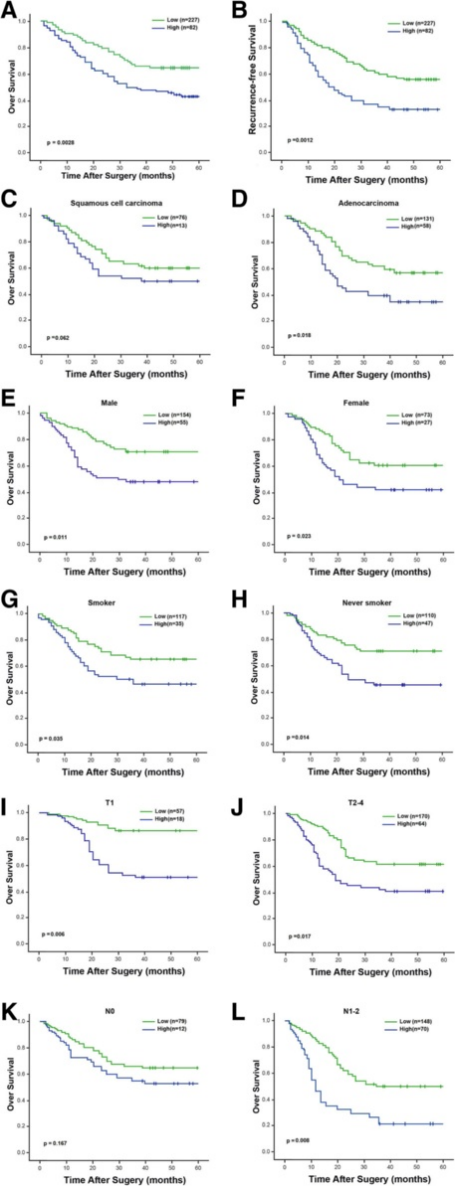
Kaplan-Meier survival analysis in patients with NSCLC. **a** and **b** Kaplan-Meier analysis of overall and recurrence-free survival according to FoxC2 expression level. **c** and **d** Subsets of patients with squamous cell carcinoma and adenocarcinoma. **e** and **f** Subsets of male and female patients. **g** and **h** Subsets of smokers and never smokers. **i** and **j** Subsets of pT status. **k** and **l** Subsets of pN status. High FoxC2 expression was associated with both shortened survival and increased recurrence in the entire cohort of patients. Subset analyses indicated that patients with high FoxC2 expression had a worse prognosis regardless of gender, smoking status and pT status. As for patients with squmous cell carcinoma and those without nodal involvement, FoxC2 was not found to be prognostic

Five-year OS and RFS rates were 46.3 % and 38.1 % respectively for the entire cohort. Patients with FoxC2 positive tumors had a significantly worse prognosis compared to those with low FoxC2 expression (OS, 43.6 % vs. 64.5 %, *p* = 0.0028; RFS, 33.4 % vs. 56.0 %, *p* = 0.0012; Fig. 2a, b). The univariate analysis indicated that FoxC2 expression, as well as TNM stage and nodal involvement had a marked influence on OS and RFS. In the multivariate analysis, FoxC2 lost its value as an independent predictor of recurrence (*p* = 0.077), although it was identified as an independent prognostic factor for OS (*p* = 0.039). Nodal status and TNM stage, two established prognostic predictors for NSCLC, remained correlated with both OS and RFS.

We then investigated the association between FoxC2 expression and prognosis, using various subset analyses. High expression of FoxC2 showed a significant correlation with poorer outcome in patients with adenocarcinoma (*p* = 0.018; Fig. 2d), whereas no prognostic impact was verified in those with squamous cell carcinoma. Patients with high FoxC2 expression had a worse prognosis regardless of gender (male, p =0.011; female, *p* = 0.023; Fig. 2e, f, respectively) or smoking status (smoker, *p* = 0.035; never smoker, *p* = 0.014; Fig. 2g, h, respectively). The prognostic value of FoxC2 expression was also examined in patients with different pT and pN status. FoxC2 was found to be prognostic in both pT1 and pT2-4 subsets, whereas could not differentiate outcome for patients without nodal involvement (pN0, *p* = 0.167; Fig. 2k). Conversely, FoxC2 demonstrated remarkable prognostic significance when combined with nodal involvement. Node-positive tumors with high FoxC2 expression displayed a much poorer outcome compared to node-positive tumors expressing low levels of the protein (pN1-2, *p* = 0.008; Fig. 2l). Thus, FoxC2 expression identifies a subgroup of patients with nodal metastasis highly prone to progression.

### High FoxC2 expression was associated with impaired E-cadhern expression and a combination of the two markers’ staining results identified a subset of patients with much worse prognosis in NSCLC

**Fig. 3:**
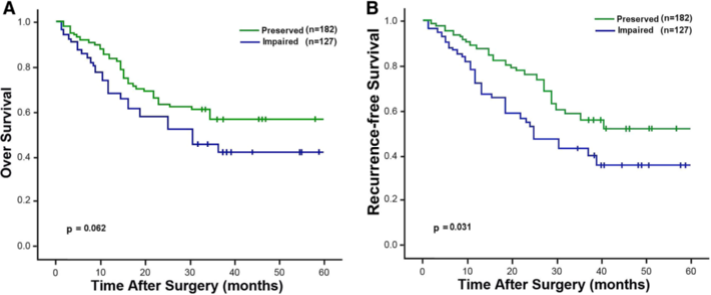
Kaplan-Meier survival analysis of overall and recurrence-free survival according to E-cadherin expression level in patients with NSCLC. **a** Overall survival; **b** Recurrence-free survival

E-cadherin expression was primarily membranous in normal and tumor cells. Impaired expression for E-cadherin was observed in 41.1 % of 309 patients (Fig. 1). Associations of E-cadherin expression with clinicopathologic characteristics are shown in Table 1. E-caherin expression exhibited no significant correlations with any of the clinicopathologic variables, except nodal involvement (*p* = 0.041). Patients with impaired E-cadherin expression had a shorter RFS compared to those with preserved expression of E-cadherin (OS, 42.2 % vs. 58.6 %, *p* = 0.061; RFS, 34.9 % vs. 55.3 %, *p* = 0.031; Fig. 3). The univariate analysis showed that E-cadherin expression had a significant influence on OS and RFS. However, E-cadherin lost its significance as an independent predictor of recurrence (*p* = 0.092) and survival (*p* = 0.098) in the multivariate analysis.

The association between FoxC2 and E-cadherin expression was also examined. A significant inverse correlation was found in both adenocarcinoma and squamous cell carcinoma (Table 2).

**Table 2:** Relationship between FoxC2 and E-cadherin expression according to pathological subtype

		Squamous cell carcinoma (*n* = 97)		Adenocarcinoma (*n* = 189)	
		FoxC2 expression		FoxC2 expression	
		FoxC2-L (%)	FoxC2-H (%)	FoxC2-L (%)	FoxC2-H (%)
E-cadherin expression	E-cadherin-L	11 (42.3)	15 (57.7)	21 (35.0)	39 (65.0)
	E-cadherin-H	65 (91.5)	6 (8.5)	110 (85.3)	19 (14.7)
*P*		<0.0001		<0.0001	

**Fig. 4:**
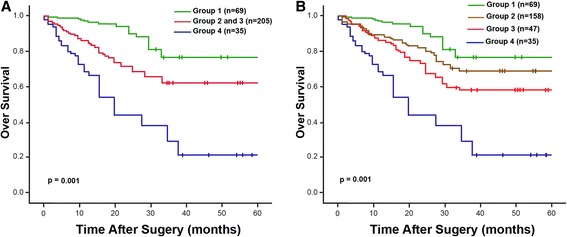
Kaplan-Meier survival analysis in patients with NSCLC according to the combination of FoxC2 and E-cadherin expression. The patients were divided into four groups: Group 1, low FoxC2 and preserved E-cadherin; Group 2, low FoxC2 and impaired E-cadherin; Group 3, high Foxc2 and preserved E-Cadherin; and Group 4, high Foxc2 and impaired E-cadherin. Patients with high FoxC2 and impaired E-cadherin expression had a much poorer prognosis compared with the other groups. **a** Group 2 and 3 were evaluated together. **b** Group 2 and 3 were evaluated separately

To investigate the accumulative effect of FoxC2 and E-cadherin expression on the prognosis of NSCLC, patients were classified into 4 groups: Group 1, low FoxC2 and preserved E-cadherin (*n* = 69); Group 2, low FoxC2 and impaired E-cadherin (*n* = 158); Group 3, high Foxc2 and preserved E-Cadherin (*n* = 47); and Group 4, high Foxc2 and impaired E-cadherin (*n* = 35). Significant differences in disease outcome were found among the 4 groups with regards to OS or RFS (Fig. 4; Table 3). Group 1 exhibited a more favorable clinical outcome (OS and RFS, 75.5 % and 62.1 %, respectively) as compared to Group 4 (OS and RFS, 20.7 % and 16.9 %, respectively), while Group2 and 3 showed an intermediate prognosis. Moreover, the multivariate analysis demonstrated that the presence of high FoxC2 expression in combination with impaired E-cadherin expression was an independent prognostic factor for shortened OS (p =0.002) and RFS (p =0.035) (Table 3).

**Table 3:** Univariate and multivariate analysis of factors associated with OS and RFS of 309 NSCLCs

	OS						RFS					
	Univariate analysis			Multivariate analysis^a^			Univariate analysis			Multivariate analysis^a^		
Variables	HR	95 % CI	*p*	HR	95 % CI	*p*	HR	95 % CI	*p*	HR	95 % CI	*p*
Age	1.50	1.01–2.89	0.092				1.12	0.62–2.36	0.147			
(≤65 vs. > 65)												
Sex	1.09	0.61–1.79	0.671				0.86	0.43–1.58	0.704			
(female vs. male)												
Tobacco use	1.26	1.17–3.08	0.143				1.03	0.52–1.97	0.766			
(no vs. yes)												
TNM stage	3.25	1.66–5.91	0.0007	2.02	0.93–4.37	0.012	3.08	1.49–6.24	0.0009	2.27	1.09–5.45	0.010
(I vs. II–III)												
pT status	1.65	0.88–2.89	0.070				1.22	0.57–2.39	0.198			
(T1 vs. T2-4)												
pN status	4.19	2.10–7.72	0.0005	2.34	1.02–5.99	0.005	3.55	1.71–7.23	0.0004	2.76	1.46–5.89	0.003
(N0 vs. N1-2)												
Pleural involvement	0.77	0.31–1.52	0.830				0.67	0.39–1.43	0.902			
(no vs. yes)												
Differentiation (well/moderate vs. poor)	1.32	0.72–2.61	0.127				1.07	0.51–1.91	0.722			
Foxc2 expression	1.98	0.75–3.92	0.036	1.90	0.76–4.05	0.039	2.13	1.08–4.15	0.011	1.54	0.89–3.67	0.077
(low vs. high)												
E-cadherin expression	2.03	0.97–4.12	0.024	1.77	0.79–3.78	0.098	2.09	1.12–4.37	0.043	1.38	0.65–2.99	0.092
(preserved vs. impaired)												
Foxc2/E-cadherin expression												
1 vs. 2	1.45	0.63–2.80	0.117				1.35	0.76–2.55	0.122			
1 vs. 3	1.70	0.88–3.23	0.062				1.47	0.71–2.68	0.103			
1 vs. 4	3.04	1.23–5.75	0.0009	2.41	1.12–4.67	0.002	2.55	1.29–5.16	0.008	2.06	0.87–4.17	0.035
1 vs. 2 + 3 + 4	1.85	0.90–3.53	0.026	1.34	0.64–2.23	0.168	1.56	0.74–3.05	0.071			

*OS* overall survival; *RFS* recurrence-free survival

^a^Variables were adopted for their prognostic significance by univariate analysis

## Discussion

The heterogeneous clinical outcomes of patients with NSCLC of the same stage lead the investigators to search for additional predictive and prognostic markers that might optimize risk-adjusted therapeutic strategies. Recent studies suggested that the activation of an EMT program in tumors may significantly contribute to disease progression. On this basis, we sought to investigate the role of FoxC2, one EMT-related molecule, in NSCLC invasiveness, as well as to evaluate its prognostic value.

Our results showed that high expression of FoxC2 significantly correlated with early recurrence and shortened survival (Fig. 2a and b). Subgroup analyses according to gender, smoking status and pT status showed consistent findings with regards to OS. In line with our results, Nishida et al. [11] reported a similar prognostic role of FoxC2 by investigating its mRNA expression in a series of 70 esophageal carcinoma cases. Analogous observation has also been recently reported for gastric carcinoma in a retrospective study of 325 patients using immunohistochemical analysis [15]. In the multivariate analysis, FoxC2 status has a prognostic impact on OS, but not RFS, independent of other prognostic factors that include node status and TNM stage. More importantly, the combined evaluation of FoxC2 with E-cadherin demonstrated independent prognostic significance in relation to both survival and recurrence (Table 3 and Fig. 3). A combination of the two markers seems to define a subgroup of patients with the worst clinical outcome within the entire cohort. This finding highlights the potential of the combination of these two molecules as a more accurate indicator in predicting disease evolution.

E-cadherin is a major cell-to-cell adhesion molecule that plays a critical role in the development and maintenance of cell polarity and tissue architecture [16]. Loss of E-cadherin expression is considered to be a hallmark of EMT and correlates with tumor invasiveness, metastasis and prognosis [17, 18]. One finding of our study is the starkly inverse association between FoxC2 and E-cadherin expression in both lung adenocarcinoma and squamous cell carcinoma. This seems to support the role of FoxC2 as a strong repressor of E-cadherin in lung cancer. The molecular mechanism behind this correlation was disclosed by the experimental study of Mortazavi et al. [19] on NSCLC cell lines, which revealed that FoxC2 can repress E-cadherin expression through downregulating p120ctn, a regulatory protein that stabilizes E-cadherin at the adhesion junctions of epithelial cells [20], by directly suppressing its promoter activity. However, an earlier study described FoxC2 as a much weaker repressor of E-cadherin in breast cancer cells [9]. This inconsistency may be attributed to the different tumor types involved in the two studies. The fact that protein expression of E-cadherin was investigated in our study while mRNA expression was observed in theirs might also contribute to this inconsistency. On the other hand, as loss of E-cadherin expression is believed to be a hallmark of EMT, the inverse correlation between FoxC2 and E-cadherin expression implicates that FoxC2 may be involved in the EMT process in lung malignancy. A subset of tumors with high FoxC2 and impaired E-cadherin status, exhibiting a stronger EMT profile, could possess more metastatic potential and have a worse prognosis, which was corroborated by our results.

We found that FoxC2 expression was heterogeneously present in lung adenocarcinoma and squamous cell carcinoma. A high level of FoxC2 expression was more frequently found in adenocarcinomas. The biological reason for this phenomenon is unknown. Nevertheless, it is comprehensible, considering the fact that gene expression profiles differ substantially between the two histological subtypes [21, 22]. Furthermore, it is interesting to note that high FoxC2 expression was closely associated with nodal involvement, as such a correlation was also shown by Watanabe et al. [23] in a study of 77 patients with extrahepatic cholangiocarcinoma. Correspondingly, the presence of FoxC2 showed a remarkable prognostic impact when combined with nodal status. Node-positive tumors with high FoxC2 expression exhibited a poorer outcome compared to those expressing low levels of the gene, whereas no such effect was found in node-negative tumors (Fig. 2k and l). Thus, FoxC2 identified a group of patients with node involvement highly inclined to disease progression. This may reflect a stronger destructive role of the protein in NSCLC with nodal metastasis, leading to a more malignant tumor phenotype.

## Conclusions

In conclusion, even though FoxC2 alone is an independent predictor for survival in NSCLC, combined evaluation of FoxC2 and E-caherin expression defines a new subgroup of patients with both shortened survival and earlier recurrence. Moreover, the assessment of FoxC2 status in node-positive patients helps identify a subset of tumors with a more aggressive phenotype. These could be of potential clinical relevance by providing new criteria for risk stratification and making these subgroups accessible for more aggressive or alternative therapy.

## Abbreviations

EMT: Epithelial-to-mesenchymal transition
NSCLC: non-small cell lung cancer
OS: overall survival
RFS: recurrence-free survival
TNM: tumor/node/metastasis
